# A novel *UGT1A1* gene mutation causing severe unconjugated hyperbilirubinemia: a case report

**DOI:** 10.1186/s12887-019-1555-y

**Published:** 2019-05-29

**Authors:** Xiaoxia Shi, Sem Aronson, Ahmed Sharif Khan, Piter J. Bosma

**Affiliations:** 1 Amsterdam UMC, University of Amsterdam, Tytgat Institute for Liver and Intestinal Research, Amsterdam Gastroenterology and Metabolism, Meibergdreef 69-71, 1105 BK, Amsterdam, The Netherlands; 2 Square Pharmaceuticals Ltd, BD, Dhaka Unit, Kaliakair, Gazipur, Bangladesh

**Keywords:** Crigler-Najjar syndrome, *UGT1A1*, HNF-1α, Genetic analysis

## Abstract

**Background:**

Crigler-Najjar syndrome (CNs) presents as unconjugated hyperbilirubinemia, as a result of UGT1A1 deficiency, and can be categorized in a severe (type I) and mild (type II) phenotype. CNs type II patients usually benefit from phenobarbital treatment that induces residual UGT1A1 activity.

**Case presentation:**

Here we present a CNs type II patient that is not responsive to phenobarbital treatment, which can be explained by two heterozygous mutations in the *UGT1A1* gene. A 3 nucleotide insertion in the HNF-1α binding site in the proximal promoter previously reported in a Crigler-Najjar patient on one allele and a novel two nucleotide deletion in exon 1, resulting in a frameshift and a premature stop codon.

**Conclusion:**

In newly diagnosed CNs patients with unconjugated bilirubin levels consistent with CNs type II but that are unresponsive to phenobarbital treatment, disruption of the HNF-1α binding site in the proximal promoter should be considered as a probable cause. Upon confirming a mutation in the HNF-1α site, phenobarbital treatment should be stopped or at least be reconsidered because of its sedative effects and its teratogenic properties.

**Electronic supplementary material:**

The online version of this article (10.1186/s12887-019-1555-y) contains supplementary material, which is available to authorized users.

## Background

Crigler-Najjar syndrome (CNs) is a rare inherited liver disorder with a severely impaired metabolism of bilirubin, resulting in the accumulation of neurotoxic unconjugated bilirubin.

This deficiency of bilirubin glucuronidation is caused by mutations in the UGT1A1 gene encoding uridine diphosphate glucuronosyl transferase, resulting in impaired enzyme activity [1]. Clinically two types of CNs are recognized. In the most severe form, CNs type I, bilirubin glucuronidation is completely lacking, while in type II, some residual activity is present. The response to phenobarbital, that induces the expression of UGT1A1 by the mediating the binding of the constitutive androstane receptor (CAR; NR1|3) to the pBREM promoter region, is used in the clinic to distinguish both forms. In this report, we describe a second patient with a serum bilirubin level normally seen in Type II that is unresponsive to phenobarbital.

## Case presentation

A 14-year old female patient from Bangladesh presented with serum total bilirubin levels around 250 μmol/L and conjugated bilirubin (measured as direct bilirubin using the Diazo method) of around 10 μmol/L, indicating a predominantly unconjugated hyperbilirubinemia. According to her parents’ description, her weight at birth was around 2000 g and 4 days after birth, her skin turned yellow. Clinical assessment revealed an unconjugated hyperbilirubinemia of 220 μmol/L without signs of erythrocyte hemolysis (major cause: ABO or Rh incompatibility). After undergoing phototherapy for 4 h a day for 4 consecutive days the serum total bilirubin levels were reduced to 153 μmol/L. The parents were advised to keep their daughter in the sunlight, but after a few months her serum total bilirubin increased again to over 300 μmol/L. From this point onward, the patient did not receive treatment and no clinical data is available because the family lives in the country side and has limited access to medical care. Between the age of 14 and 17 years her serum total bilirubin levels have been monitored and where stable around 200–250 μmol/L. Liver damage markers in serum were low (ALT) and a hemolytic cause of the hyperbilirubinemia was excluded with normal hemoglobin and reticulocyte levels (Table 1). These serum bilirubin levels without any treatment are in line with those seen in Crigler-Najjar syndrome (CNs) type II, indicating a partial deficiency of UGT1A1. However, inducing the residual UGT1A1 activity by administrating phenobarbital (30 mg/day) did not result in a significant change in total bilirubin (Table 1). The coding region and intron-exon boundaries of the UGT1A1 gene of the patient and parents was sequenced to determine what caused UGT1A1 deficiency and the absence of a response to phenobarbital.

**Table 1:** Laboratory results between 2015 and 2018 show an unconjugated hyperbilirubinemia without signs of liver damage or hemolysis

Date	Test	Result	Unit
29/08/2018	Total bilirubin	241	μmol/L
	Albumin	36	g/L
15/10/2017	Total bilirubin	251.4	μmol/L
	Direct bilirubin	10.2	μmol/L
20/12/2016	Total bilirubin	217.6	μmol/L
	Albumin	46	g/L
24/05/2016	Total bilirubin	255	μmol/L
23/03/2016	Total bilirubin	214.2	μmol/L
	Alanine aminotransferase (ALT)	22	U/L
27/12/2015	Total bilirubin	210.7	μmol/L
	Direct bilirubin	1.7	μmol/L
	Alkaline Phosphatase (ALP)	87	U/L
	Gamma-GT (ɣ-GT)	18	U/L
21/12/2015	Hemoglobin	12.20	g/dL
	Reticulocyte	0.86	%
20/12/2015	Total bilirubin	247	μmol/L
	ALT	21	U/L

### Sequencing of the UGT1A1 gene coding and promoter region

Sanger sequencing was used to determine the nucleotide sequence of amplified fragments of UGT1A1 gene and promoter region as previously described [2, 3]. The purified amplicons were sequenced using the internal primers listed in Additional file 1: Table S1.

### Genetic analysis of the UGT1A1 gene and promoter

**Fig. 1:**
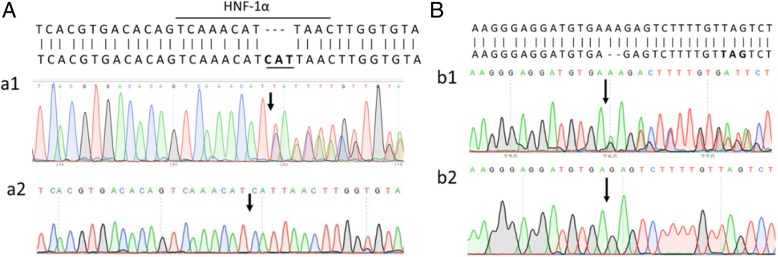
Sequencing chromatograms of the patient and her parents. The row in each chromatograms indicated the position of the identified mutation. **a** The sequence of normal bases in this specific part of the UGT1A1 gene was shown in the upper row, whereas the matched lower row showed the bases (CAT) that were inserted in the patient (a1) and her mother (a2). **b** The sequence of normal bases in this specific of the UGT1A1 gene is shown in the upper row, whereas the matched lower row showed the deleted bases (AA) in the patient (b1) and her father (b2)

The patient was found to have two heterozygous mutations in the *UGT1A1* gene (Fig. 1). The first is a 3 nucleotides insertion in the HNF-1α binding site in the proximal promoter, which was also found in her mother. The second mutation is a two nucleotides deletion in exon 1 (266_267delGT), which was found in her father. This two nucleotide deletion has not been described in literature before and results in a frameshift with a predicted premature stop codon at position 279_281TAG in exon 1.

## Discussion and conclusion

Here we present a patient with an unconjugated serum bilirubin level reported for patients with CNs type II [4], that does not respond to phenobarbital treatment. The patient appeared to be heterozygous for two different *UGT1A1* mutations.

The mutation derived from her mother, a 3 nucleotide insertion in the HNF-1α binding site in the proximal promoter, was identical to the mutation we found in a CNs patient with a similar phenotype that we reported recently, this 3 nucleotide insertion caused a strong reduction of basal promoter activity (− 95%) and made the promoter non-responsive to CAR activation and to a potential alternative treatment via PXR activation with rifampicin which were confirmed by the functional promoter studies in that report. Due to this mutation, a minute amount of mRNA will be transcribed from this allele, which encodes for a minimal amount of normally active protein, and renders this allele unresponsive to *UGT1A1* inducing drugs, including phenobarbital treatment.

The two nucleotide deletion in exon 1 of the *UGT1A1* gene, inherited from her father, is a novel mutation. This mutation has not been described before and is predicted to result in a premature stop codon by frameshift leading to the formation of a truncated and inactive enzyme that will most likely be degraded.

The combination of these two mutated alleles results in a severely impaired UGT1A1 function, which is in line with the high levels of unconjugated hyperbilirubin seen in this patient. Unconjugated bilirubin levels in serum of this patient were comparable to that seen in the previous patient with an identical HNF-1a mutation, indicating the phenotype results from this mutation only. Since, the HNF-1α mutation renders the gene unresponsive to transcriptional activation of the *UGT1A1* gene by phenobarbital, phototherapy and liver transplantation are currently the only therapeutic options.

We report a second patient with a CNs type II phenotype that is unresponsive to phenobarbital treatment due to a mutated HNF-1α binding site, in combination with a novel nonsense mutation. In newly diagnosed CNs patients with a similar phenotype, looking for mutations in the HNF1a binding site seems a good strategy. Upon confirming presence of a mutated HNF-1a binding site the use of phenobarbital should be reconsidered in view of its sedative effect and specifically in women, because of its reported teratogenic properties [5].

## Additional file

Additional file 1: **Table S1.** Primer used to amplify *UGT1A1* gene*.* (DOCX 18 kb)

## Data Availability

All used data and materials are available upon request, please contact P.J. Bosma.

## Abbreviations

ALT: Alanine transaminase
CNs: Crigler-Najjar syndrome
HNF-1α: Hepatocyte nuclear factor 1α
*UGT1A1*: UDP-glucuronosyltransferase 1 A1
