# Supplementary material for: A novel UGT1A1 gene mutation causing severe unconjugated hyperbilirubinemia: a case report
Source: BMC Pediatr. 2019 May 29;19:173. doi: 10.1186/s12887-019-1555-y (PMC6540546; doi:10.1186/s12887-019-1555-y)
Supplement: Supplementary file 1 — Table S1. Primer used to amplify UGT1A1 gene. (DOCX 18 kb) [file 12887_2019_1555_MOESM1_ESM.docx]

Additional file 1: Table S1

| PRIMER | FORWARD PRIMER | REVERSE PRIMER |
| --- | --- | --- |
| UGT1A1 proximal promoter (sequence) | GTCTGGCTCACCTCATGGC | CGTCAGGTGCTAGGACAACTA |
| UGT1A1 gtPBREM region (sequence) | TAACCTGAAACCCGGACTTGG | CATGCTATCACTCAGGTGCC |
